# Harmonizing self-reported and free text medication data: a reproducible pipeline for gerontological research

**DOI:** 10.1186/s12911-025-03332-w

**Published:** 2025-12-31

**Authors:** Ramkrishna K. Singh, Chen Chen, Semere Bekena, David C. Brown, Kaylin Taylor, Matthew Blake, Yiqi Zhu, Kebede Beyene, David B. Carr, Ganesh M. Babulal

**Affiliations:** 1 Department of Neurology, Washington University School of Medicine in St. Louis, 600 S. Taylor Avenue, Suite 111Z, St. Louis, MO 63110 USA; 2 Institute of Public Health, Washington University School of Medicine, St. Louis, MO USA; 3 Evernorth Health Services, St. Louis, MO USA; 4 Department of Medicine, Washington University School of Medicine, St. Louis, MO USA

**Keywords:** Medications, Gerontology, Dementia, Natural language processing, Electronic health records, Data curation

## Abstract

**Background:**

Self-reported medication data collected as free text in gerontological and dementia research is often unstructured with inconsistent formatting. These circumstances pose a challenge for standardization and classification when preparing effective, reproducible analyses. Spelling variations, naming conventions, and reporting drug combinations can hinder mapping to standard pharmacologic vocabularies and compromise medication exposure assessments. We aimed to develop and implement a transparent, reproducible, and scalable data harmonization pipeline that ingests free-text medication records and classifies them according to American Hospital Formulary Service (AHFS) therapeutic categories.

**Methods:**

A four-phase curation pipeline processed 30,062 Research Electronic Data Capture (REDCap) medication records collected over nearly a decade of annual visits in The Driving Real-world In-Vehicle Evaluation System (DRIVES)Project. In Phase 1, the pipeline standardized medication names using deterministic and fuzzy matching techniques, incorporating Drug-Named-Entity Recognition (DER), the *thefuzz* Python library, and expert review. Phase 2 mapped drugs to AHFS categories via DrugBank and RxNorm. Phase 3 generated a wide-format dataset with binary class-level exposure indicators. Phase 4 involved a final quality review with auditable documentation.

**Results:**

Out of 30,062 entries, 16,902 eligible prescription entries remained after the removal of vitamins, supplements, and over-the-counter (OTC) drugs. Of these, automated or semi-automated processes successfully standardized 94.2% of entries, with only 5.8% requiring further expert review. A total of 444 unique medications were successfully mapped to AHFS classifications. The curated dataset enables efficient integration into analytical models and supports reproducible assessment of medication exposure.

**Conclusions:**

This pipeline addresses a key methodological challenge in clinical research by providing a reproducible, scalable solution for harmonizing unstructured medication data and enhancing its analytical utility.

**Supplementary Information:**

The online version contains supplementary material available at 10.1186/s12911-025-03332-w.

## Background

Medication data is a cornerstone of clinical research, yet in practice, these data are either not collected or reported or collected in formats that are inconsistent, incomplete, or loosely structured [1]. Electronic data capture platforms, such as Research Electronic Data Capture (REDCap), are widely used for manually recording patient medication histories in prospective, clinical, and registry-based studies due to their flexibility and ease of deployment [2–5]. However, this flexibility brings significant challenges. Free text entries introduce variability in capitalization (e.g., “lipitor” vs. “Lipitor”), spelling errors (e.g., “amitriptiline” vs. “amitriptyline”), inconsistent use of brand or generic names (“acetaminophen” vs. “Tylenol”), difficulty capturing combination products (e.g., “lisinopril-hctz” vs. “lisinopril/HCTZ 20-12.5 mg”), along with varying dosages and frequencies [6, 7]. These factors complicate automated mapping to standard vocabularies (e.g., Normalized Names for Clinical Drugs [RxNorm], Anatomical Therapeutic Chemical classification system [ATC], and American Hospital Formulary Service [AHFS]) and reduce the accuracy of drug-exposure coding. For example, studies show a misspelling rate of up to 17% in electronic health records (EHR) free-text medication entries [8, 9], with uncorrected errors reducing the trustworthiness of research data and its reproducibility. Accurate medication capture has become even more challenging when studying older adults who have an ever-increasing level of polypharmacy. Even with specific data quality rules, the problem posed by free text is especially pronounced for longitudinal research, multi-site/source/protocol studies, and legacy applications or computer systems.

Poor-quality drug data undermines critical clinical and epidemiologic analyses. Drug exposure phenotyping used to glean patterns of medication use, such as statin or antihypertensive exposure, relies on accurate identification of prescribed agents [10, 11]. Data noise from misspellings or incomplete names can misclassify exposure status and bias results. Similarly, pharmacovigilance efforts aimed at detecting adverse drug events or drug-drug interactions depend on precise medication labels; [12, 13] mislabeling can mask harm signals or generate false positives [14]. 

Polypharmacy is universal in gerontological cohorts, especially studies on dementia. Standard drug burden indices reveal that many patients with dementia are on more than fivemedications and receive at least one potentially inappropriate medication [15–18]. High medication loads not only increase the risk of functional decline [19, 20] but also complicate documentation: distinguishing statins from supplements, establishing drug classes, and assessing cumulative drug risk becomes difficult without systematic mapping. Incorrect cataloging of drug classes may either underestimate or exaggerate this burden. Moreover, comparative effectiveness research, especially across sites or registries, requires consistent medication definitions; otherwise, site-specific documentation practices lead to poor reproducibility and biased inter- and intragroup comparisons [21–23]. 

Classification issues are especially critical in multi-site trials and chronic disease cohorts. In registry-based dementia studies, inconsistent medication captured across centers hinders the conduct of meta-analyses and the generalizability of findings [24]. While platforms like REDCap have improved scalability and accessibility of clinical data collection, they also underscore the need for complementary data harmonization pipelines to address inconsistencies inherent in free text entries and enhance downstream analytical utility.

Prior studies have primarily addressed medication harmonization within structured EHR systems, emphasizing the use of controlled vocabularies such as RxNorm, the Anatomical Therapeutic Chemical (ATC) classification, or the National Drug File–Reference Terminology (NDF-RT) [25–29]. These frameworks rely on predefined medication lists, facilitating straightforward mapping. However, they are less applicable to unstructured, self-reported data collected via Research Electronic Data Capture (REDCap), which often includes misspellings, brand–generic inconsistencies, and incomplete medication details [30]. In such datasets, participants may not always be familiar with their medications, leading to additional variability. Our pipeline directly addresses this challenge by reducing classification errors, retaining maximum usable data, and enhancing research validity without discarding ambiguous entries. This reproducible, open-source framework is particularly tailored to gerontological and dementia research, where polypharmacy and caregiver-reported medication lists are common.

Medication data quality issues are magnified among older adults and those with cognitive impairment. First, analysts often rely on subjective recall when medication histories are not linked to pharmacy fill records or EHRs [31, 32]. Recall bias, a well-documented phenomenon, particularly in aging studies, can skew data when patients fail to remember their medications or report incorrect doses [33]. In dementia populations, this issue is amplified by memory loss; thus, caregiver proxy reporting is commonly used. Yet, caregiver reports are subject to their own inaccuracies and may be incomplete [34, 35]. 

Second, medication adherence can be erratic due to affordability constraints (e.g., co-payments) [36, 37], physical access limitations (living far from pharmacies) [38], or confusion over the drug’s purpose, making it difficult to distinguish between what patients consumed versus what was prescribed [39, 40]. Documenting drug exposure materially affects accurate interpretation in clinical trials (e.g., a participant “on” donepezil but not taking it cannot be considered exposed).

Memory impairment, polypharmacy, affordability, and barriers to access compound to create an environment where medication histories captured in research platforms are prone to misclassification, jeopardizing clinical inferences in dementia research.

Efforts to standardize medication data often leverage established classification systems, including the ATC system, RxNorm, or the AHFS Pharmacologic-Therapeutic Classification System [41, 42]. While these frameworks offer robust pharmacologic and therapeutic hierarchies, real-world data frequently fails to map directly due to input inconsistencies, the use of abbreviations, or missing dosage information [43]. Furthermore, such systems may not align directly with the analytic requirements of a specific study, particularly when therapeutic effects are distributed across pharmacologically related subclasses.

Large datasets often contain hundreds of distinct medications, which can impact analyses when converted to high-dimensional, sparse binary matrices. This fragmentation can limit statistical power and obscure shared mechanisms of action or therapeutic indications [44]. Merging medications into broader pharmacologic groups can improve interpretability, reduce sparsity, and support class-level inference. However, such consolidation efforts must be conducted transparently and systematically to ensure analytical reproducibility.

This study presents a generalizable, reproducible, rule-based protocol for curating and classifying real-world clinical or research medication data collected via REDCap. Our approach employs a structured four-phase pipeline: drug name standardization using both programmatic and manual methods; hierarchical mapping to AHFS categories; logical merging of pharmacologically related drug subclasses; and a final quality review. The resulting dataset includes binary therapeutic class indicators, supporting scalable analysis of medication exposure.

## Methods

Medication data were reported by participants enrolled in The Driving Real-world In-Vehicle Evaluation System (DRIVES) Project, a longitudinal study that was established in 2014 at Washington University School of Medicine in St. Louis [45]. The primary wave of participants, who were cognitively normal (Clinical Dementia Rating [CDR] = 0), adults aged 65 years or older, were referred from the longitudinal Memory and Aging Project (established in 1979) and the Knight Alzheimer’s Disease Research Center (established in 1984). Participants completed annual cognitive assessments during structured annual office visits. Medications were self-reported using structured REDCap forms. Participants confirmed medications at home or brought medication lists and/or containers (“brown bag”) to improve reporting accuracy. Trained clinical research coordinators processed the self-reports, recording all medications taken in the past year, including name, dosage, route, frequency, and start date in a REDCap instrument [46, 47]. The Washington University Human Research Protection Office approved all procedures under Institutional Review Board (IRB) protocol identifiers 201,706,043, 202,010,214, and 202,003,209, which were conducted in strict accordance with the 1964 Declaration of Helsinki.

We developed a structured four-phase data curation pipeline to standardize and classify free-text medication entries from REDCap into structured binary indicators using the American Society of Health-System Pharmacists (ASHP)’s AHFS [48] Pharmacologic-Therapeutic Classification System acquired through an annual DrugBank Academic + License [49, 50]. The pipeline was implemented in Python 3.12 using the Drug-Named-Entity-Recognition (DER) library (v2.0.0) [51], and *thefuzz* (v0.22.1) [52] for fuzzy matching. The raw medication data were collected from February 2008 to February 2025. Data processing, including cleaning, standardization, and harmonization, was conducted between November 2024 and February 2025.

### Phase 1: drug name standardization

The initial phase involved cleaning and correcting the raw medication entries. First, we narrowed our focus to only prescription medications by excluding over-the-counter (OTC) drugs, vitamins, and supplements using a list compiled by subject matter experts. Then, we corrected and standardized the medication names using the DER library. We applied an absolute matching algorithm using the *find_drugs()* function to map each raw REDCap medication entry to a standardized drug name. For example, the function can be represented as *find_drugs(“fluoxetine”. split(“ ”)*,* is_ignore_case = True). In practice*,* it was executed iteratively for each medication entry using that entry’s own tokenized text.* This operation yielded three datasets: (i) correct matches (accepted as standardized), (ii) ambiguous matches (held-out entries with multiple potential matches, requiring manual validation), and (iii) unmatched entries. Unmatched entries, those with no acceptable match, were automatically forwarded to the next matching algorithm. Ambiguous entries, defined as cases returning multiple matching drug names, were not processed further in the automated pipeline. Instead, they were flagged and stored in a separate ambiguous-match queue and resolved only during the final study-affiliated pharmacist–physician adjudication step. This ensured that only high confidence matches advanced through the automated layers while preventing uncertainty from propagating across phases.

**Fig. 1:**
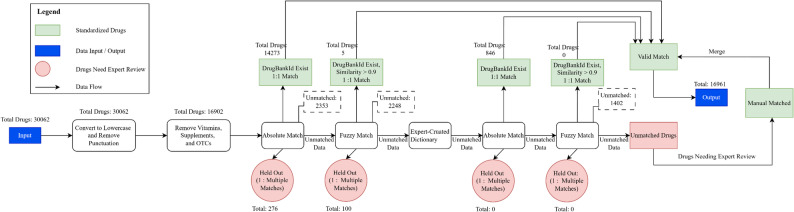
Workflow for Medication Name Standardization and Preprocessing. (**a**) OTC = Over the Counter. (**b**) Light green-shaded nodes indicate successfully standardized drugs. (**c**) Light red-shaded nodes represent entries requiring expert input. (**d**) Blue-shaded nodes represent the input or output data. (**e**) Arrows denote the direction of data flow

Subsequently, fuzzy matching using function *find_drugs(raw medication entry.split(“ ”)*,* is_ignore_case = True*,* is_fuzzy_match = True)* with spelling tolerance was applied to each unmatched entries iteratively using a similarity threshold of 0.90 (≥ 90% similarity, determined through expert review of 106 fuzzy-matched results) to recover additional valid mappings while optimizing for match sensitivity and specificity. The similarity was computed using the Jaccard index based on overlapping character n-grams between strings. A reusable reference dictionary of brand and generic names, developed during initial setup, resolved remaining entries. Unmatched cases, particularly those with a similarity score below 90%, were independently reviewed by one study-affiliated pharmacist and two physicians. Given the varying levels of granularity within AHFS classifications, expert input was necessary to ensure a clinically meaningful mapping for medications. However, manual input was limited to the pipeline’s initial development. Experts codified their decisions systematically in an auditable log and resolved terms were integrated into the reusable drug dictionary, iteratively reducing manual review requirements as subsequent entries were added to the dataset. The stepwise process for standardizing free-text medication names is shown in (Fig. 1). A detailed summary of the most common data entry issues encountered during medication name standardization is provided in Supplementary Table 1.

### Phase 2: therapeutic class mapping

In the second phase, only unambiguous Phase 1 outputs proceeded to therapeutic class mapping. All ambiguous entries were withheld for manual review.We standardized medications by mapping records to AHFS Pharmacologic-Therapeutic classifications using reference lists from DrugBank (DrugBank Online version 5.1.13, Academic + License) [49, 50]. Standardized medication names were first matched using an exact deterministic string-matching approach against the AHFS list of generic drug names. Each entry, normalized for case and punctuation, was compared character-for-character with AHFS reference terms, and records achieving 100% text equivalence were automatically classified as direct matches (e.g., ‘atorvastatin’ → Atorvastatin; ‘Lipitor’ → Lipitor). Subsequent remaining unmatched medications were then matched using the *extractOne()* function from *thefuzz* Python package, which applied the Levenshtein distance algorithm, with a similarity threshold of ≥ 90%, benchmarked against an RxNorm Semantic Clinical Drug Form description. The initial similarity threshold was set at 86%, based on empirical validation through expert review of 15 randomly sampled matches distributed across three similarity ranges ([86–90], [90–95], and [95–100]). To improve precision and minimize false positives, the final threshold was increased to 90%. The fuzzy matching threshold of 90% was selected after pilot testing different similarity levels (85%, 90%, and 95%) on a small subset of medication entries to identify the value that produced the most accurate matches with minimal errors. This threshold proved effective for our dataset, which exhibited moderate variation in spelling and formatting. In practice, this calibration is a one-time process conducted during the initial setup of the pipeline. Once an optimal value is established, it can be consistently applied to all future data from the same source. For datasets with more frequent misspellings, a lower threshold (e.g., 85%) may capture more valid entries, while cleaner datasets may benefit from a higher threshold (e.g., 95%) to minimize false matches. Adjusting this setting at the outset ensures reliable and reproducible performance across diverse datasets.

**Fig. 2:**
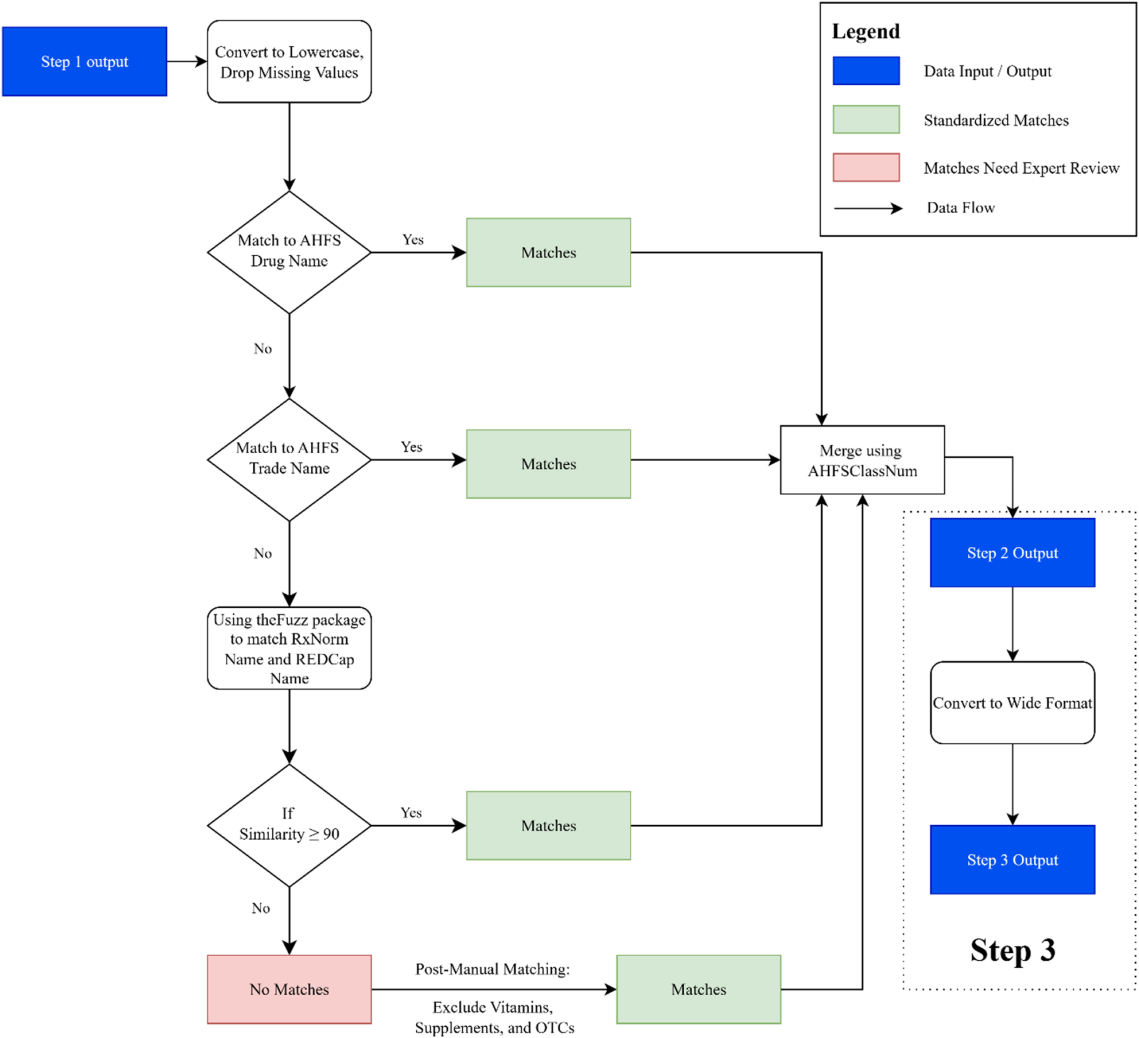
Workflow for Mapping Standardized Drug Names to AHFS Therapeutic Classes. (**a**) The initial dataframe matched to AHFS generic drug names was merged with the dataframe matched to AHFS trade names using the unique key “UN.” The resulting dataframe was then merged with the tblAHFSClass using “AHFSClassID.” Next, the merged dataframe was linked to tblAHFSClass_Extended using “AHFSClassNum.” The dataframe match with mapAHFSClass_SCDF was also merged with tblAHFSClass_Extended via “AHFSClassNum”. (**b**) AHFS, American Hospital Formulary Service; REDCapName, standardized variable name used in REDCap database; AHFSClassNum, Unique identifier for AHFS drug classification; *thefuzz*, Python’s fuzzy string-matching package used for approximate text matching. (**c**) Matching performed sequentially through AHFS drug name, AHFS trade name, and fuzzy string comparison with a similarity threshold of ≥ 90. (**d**) Expert-curated dictionaries used to finalize unmatched entries

**Fig. 3:**
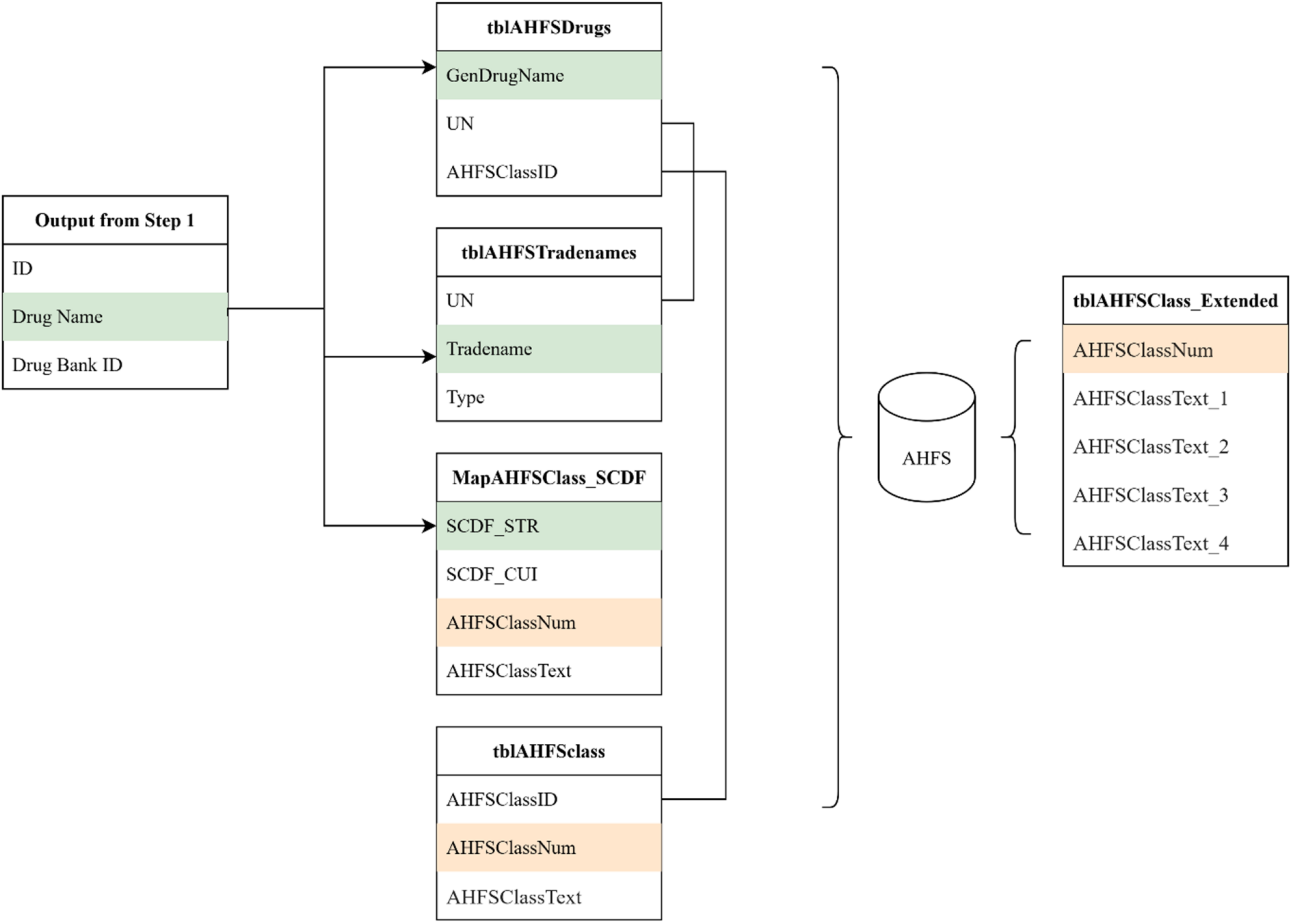
Relational Structure of AHFS Tables Used to Map Standardized Drug Names to Pharmacologic and Therapeutic Classes. (**a**) Drug Name: Any drug term found in AHFS for this monograph. (**b**) GenDrugName: This is the short title of an AHFS Drug Information monograph. (**c**) Tradename: Any drug term found in AHFS for this monograph. (**d**) SCDF_STR: The RxNorm Semantic Clinical Drug Form description. (**e**) SCDF_CUI: The RxNorm Semantic Clinical Drug Form concept unique identifier. (**f**) AHFSClassNum: The classic AHFS classification number (e.g., 08:12.06.04). (**g**) AHFSClassText: The full descriptive name of the AHFS classification. (**h**) AHFSClassText_2: Second-level AHFS class (first 4 digits). (**i**) AHFSClassText_3: Third-level AHFS class (first 6 digits). (**j**) AHFSClassText_4: Fourth-level AHFS class (full 8-digit hierarchy)

Entries still unmatched after this step progressed to manual classification. During pipeline calibration, unmatched medication entries were manually reviewed by a study-affiliated pharmacist and physician. Their input, limited to this first phase, was guided by documented, clinically informed decision rules. In addition to resolving unmatched terms, these experts also served as a quality control checkpoint, ensuring the clinical appropriateness of therapeutic class assignments, particularly given the complexity and varying levels of specificity in AHFS classifications. This review was especially important for medications relevant to gerontology and dementia. All decisions were recorded in an auditable log and used to inform automated classification in future datasets, thereby minimizing the need for further manual review. The end-to-end workflow for mapping standardized drug names to AHFS classes, along with the underlying relational database structure supporting this process, is illustrated in (Figs. 2 and 3). A complete list of therapeutic classes, along with their mapped medications, is provided in Supplementary Table 2.

### Phase 3: dataset transformation

Following the therapeutic class assignment, the medication data were initially transformed into a long format, where each row represented a medication-class pairing per participant per visit. This format was then transformed into a wide format, in which each row represented a participant with binary indicators for each AHFS therapeutic class at each visit. Each indicator denoted the presence (1) or absence (0) of medications within that class. This wide-format structure enhances analytic clarity by consolidating heterogeneous free-text entries into standardized therapeutic-class indicators that can be readily incorporated into statistical models. Rather than referring to model-level interpretability as used in informatics and NLP our use of the term reflects improved human comprehensibility of the medication exposure variables themselves. For example, multiple medications such as simvastatin, atorvastatin, and rosuvastatin are collectively represented under the single AHFS class ‘Antihyperlipidemic Agents’. This reduces dimensionality, minimizes data sparsity, and enables clinically meaningful class-level analyses (e.g., evaluating lipid-lowering drug exposure) while maintaining consistency across visits and participants. A reusable mapping dictionary, developed during pipeline calibration, ensured consistent and reproducible therapeutic-class assignment across datasets.

### Phase 4: final quality review

In the final phase, study-affiliated experienced physicians conducted a quality assurance review. This involved randomly verifying the accuracy of medication trade names, generic names, ingredient compositions, and their corresponding AHFS classifications. The goal was to ensure that the final therapeutic class assignments were both pharmacologically appropriate and aligned with the study’s research focus. All evaluations followed predefined internal protocols, which included random sampling procedures, dual verification for complex cases, and use of established drug references such as DrugBank guides. Each step was documented in a structured audit log to ensure transparency, reproducibility, and the overall integrity of the dataset (Supplementary Table 3, Sections 1–3). The complete medication matching code is available on GitHub, with the access link provided in (Supplementary Appendix 1).

## Results

The medication data curation pipeline was applied to 30,062 raw medication entries collected via REDCap from participants in the DRIVES. These entries included 4,990 unique free-text medication names prior to standardization. Initial normalization, converting all text to lowercase and removing punctuation, reduced the number of unique names to 3,707. Application of a curated exclusion list to remove vitamins, supplements, and OTC medications resulted in 16,902 medication entries comprising 2,575 unique names eligible for downstream processing.

### Phase 1: drug name standardization

Of the 16,902 eligible entries, 14,273 (84.4%) were successfully standardized to generic drug names via exact matching using the DER library (Table 1). An additional 276 entries were flagged for manual review due to an ambiguous one-to-many (Phase 1, Process 1: Correct = 14,273; Ambiguous = 276; Unmatched = 2,353). Fuzzy string matching, using a similarity threshold of ≥ 90%, was applied to 2,353 unmatched entries and resolved five (5) additional high-confidence matches. A further 100 borderline entries, defined as those with a fuzzy match similarity score between 85% and 90% were held for manual review due to their uncertain or potentially misleading partial matches (Phase 1, Process 2: Correct = 14,278; Ambiguous = 376; Unmatched = 2,248).

**Table 1:** Number and percentage of drug names retained for each processing steps

Phase	Process	Correct Matches	Ambiguous Matches	Unmatched	Total	Percentage
Phase 1: Drug Name Standardization	1. Absolute Match	14,273	276	2353	16,902	84.4%
	2. Fuzzy Match (*DER* library)	5	100	2248		0.03%
	3. Absolute Match Through Expert-Cruated Dictionary	846	0	1402		5.0%
	4. Fuzzy Match Through Expert-Cruated Dictionary	0	0	1402		0%
	5. Expert Review	1778	0	0		10.5%
Phase 2: Therapeutic Class Mapping	1. Direct Matching to AHFS Generic Names	389	NA	88	477 (unique)	81.6%
	2. Matching AHFS Trade Names	31	NA	57		6.5%
	3. Fuzzy Match (*thefuzz* library)	19	NA	38		4.0%
	4. Expert Review	38	NA			8.0%

a. Ambiguous matches: entries with one-to-multiple potential standardized drug names (represented as red shaded nodes in Fig. 1)

b. Unmatched entries: entries with no identified standardized drug names

c. Percentage: number of correct matches divided by total number of entries at each phase

Out of the remaining 2,248 unmatched medication entries, 846 were resolved through absolute and fuzzy matching using the known brand and generic drug name variants (Phase 1, Process 3&4: Correct = 15,124; Ambiguous = 376; Unmatched = 1,402). The final 1,402 entries (8.3% of the cleaned dataset) underwent manual review by a study-affiliated pharmacist and physician. Both independently classified the unmatched medications by reviewing generic names, trade names, and mechanism of action. Following their individual reviews, the pharmacist and clinician conducted joint review sessions to compare assignments, resolve discrepancies, and ensure consistency in therapeutic class mapping and AHFS classification. The research focus guided all decisions, the clinical appropriateness of categorization, and the granularity of the AHFS system. This review process was fully documented in a structured audit log, and adjudicated terms were incorporated into a reusable drug dictionary to facilitate automated resolution of similar entries in future datasets without repeated expert review. This process resulted in 16,961 fully standardized medication entries, with 94.2% resolved via automated or semi-automated procedures and 5.8% through expert adjudication.

### Phase 2: therapeutic class mapping

Following name standardization and removal of duplicates and missing values, 477 unique drug names remained for therapeutic classification. Direct matching to AHFS generic names resolved 389 entries (81.6%) (Phase 2, Process 1: Correct = 389; Unmatched = 88).

An additional 31 unmatched entries were resolved using AHFS trade names, and 19 more via fuzzy matching at the 90% similarity threshold (Phase 2, Process 2&3: Correct = 439; Unmatched = 38). The final 38 unmatched medications were manually classified using pre-specified decision rules and clinical expert review.

In total, 439 of the 477 medications (92%) were classified using automated or semi-automated processes, with 38 resolved via expert input. All AHFS assignments were recorded in a persistent classification map to ensure consistency and reusability. The final curated dataset included 444 unique medications with valid AHFS assignments, each linked to the complete four-level AHFS hierarchy. A quality assurance review was conducted to validate classification accuracy, identify overlapping categories, and consolidate pharmacologically similar classes. These refinements were implemented according to a documented correction protocol and were uniformly applied across the dataset.

### Phase 3: dataset transformation

The transformed dataset yielded binary indicators of medication use for each AHFS pharmacologic class across visits. These indicators allowed for precise quantification of medication exposures. The use of a standardized mapping dictionary ensured consistent classification and enabled reproducible application of the pipeline.

### Phase 4: final quality review

A structured quality review, conducted by study-affiliated physicians, was completed to confirm the reliability of the curated dataset. Randomly sampled entries were assessed for consistency between medication names and their assigned AHFS classifications. No discrepancies were identified, and all reviewed assignments were deemed pharmacologically appropriate and aligned with the study’s objectives. The audit log provided traceable documentation for all verification steps, supporting the overall integrity and reproducibility of the final dataset.

## Discussion

Our primary objective was to construct a scalable and transparent pipeline that transformed unstructured medication entries into structured, analyzable variables mapped to a standardized pharmacologic hierarchy. By combining automated tools, including exact and fuzzy matching, with one-time structured expert review and classification via the AHFS, we achieved 94.2% standardization through automated or semi-automated methods, while only 5.8% required manual intervention. The resulting dataset includes binary indicators of medication use by therapeutic class, allowing for reproducible and interpretable analyses of medication exposure. This work addresses a critical methodological gap in prospective research and registry studies that rely on REDCap or similar research electronic data capture platforms, where unstructured medication data often limits downstream analysis and introduces misclassification error [5]. 

A key contribution of this work is in demonstrating how a multi-phase, logic-driven approach can systematically resolve the ambiguity and variability inherent in free-text medication data commonly encountered in clinical or research workflows. Although REDCap provides flexibility and ease of deployment, its lack of structured medication entry fields frequently results in idiosyncratic naming conventions, misspellings, and inconsistent use of brand versus generic names [5, 53, 54]. Without systematic curation, these issues reduce the reliability of medication exposure definitions and compromise analytic validity. Our pipeline directly addresses these limitations by integrating automated natural language processing (NLP), fuzzy matching, and limited manual matching. Unlike prior EHR-based harmonization methods that depend on structured inputs or proprietary tools [25–29], our pipeline processes self-reported, free-text medication data using open-source natural language processing and fuzzy string-matching algorithms combined with expert adjudication. This approach enables reproducible harmonization across interdisciplinary teams and supports transparent, scalable research in aging and dementia populations.

Although clinical expert review was incorporated during initial development to confirm the accuracy of ambiguous mappings, this step serves as a calibration procedure rather than a required component of routine use. The harmonization pipeline is designed to function autonomously through deterministic and probabilistic matching algorithms, supported by a reusable reference dictionary. In settings where expert review is not feasible, investigators can validate automated mappings through random sampling or cross-referencing with open pharmacological resources, such as DrugBank or RxNorm. It has important implications for free-text EHR medication harmonization, where scalable, automated, and reproducible solutions are necessary to efficiently process large volumes of unstructured medication data. This flexibility ensures scalability and reproducibility across studies, even when domain experts are not directly involved in the process.

We also found that combining exact and fuzzy matching approaches improved standardization efficiency without sacrificing accuracy. Specifically, we utilized the DER library for deterministic matching and the fuzz string similarity metrics to resolve near matches, with an empirically defined threshold to minimize false positives. This approach reduced the volume of records requiring manual intervention and optimized the trade-off between scalability and precision. The incorporation of key expertise (pharmacy, internal medicine, geriatrics) in the review process further enhanced the resolution of unmatched entries, especially those involving brand or generic mismatches or common spelling variants. Similar hybrid NLP-manual frameworks have demonstrated success in prior studies of clinical text standardization, serving as a model for real-world data environments where input variability is high [27, 55, 56]. 

Both analytic and clinical considerations guided the decision to adopt the AHFS Pharmacologic-Therapeutic Classification System. AHFS provides a four-level hierarchy that allows for grouping medications based on pharmacologic mechanism and therapeutic use [41, 42]. This structure facilitates class-level inference, which is particularly useful in aging research where exposure to drug classes rather than individual agents is often of interest. While other classification systems, such as RxNorm or the ATC classification, are widely used, they either emphasize chemical structure and dosing form (RxNorm) or employ a structure that is less intuitive for United States-based clinical research [41, 42]. 

Our work builds on the prior studies, which emphasized the need for standardized approaches to medication data classification [41, 57]. While prior efforts primarily focused on structured EHR data and integration with vocabularies like RxNorm and National Drug File-Reference Terminology (NDF-RT), they offer limited solutions for registry and cohort studies that rely on unstructured, self-reported medication inputs, often lacking controlled vocabularies or autocomplete features [58]. To address this gap, we developed a transparent, replicable pipeline tailored to REDCap-based studies. Unlike most existing tools, which are often proprietary, narrowly scoped, or not easily generalizable, our approach uses open-source Python libraries, a publicly available drug classification system, and a validated adjudication protocol to ensure scalability and broad applicability. The pipeline also addresses the high dimensionality of real-world medication datasets by collapsing free-text entries into binary indicators of therapeutic class. This reduces data sparsity and supports more stable epidemiologic modeling by mitigating multicollinearity and low event-per-variable ratios. The resulting wide-format structure integrates smoothly with other covariates, facilitates variable selection, and enhances reproducibility across multi-site studies.

This study has several strengths. First, it features a transparent and modular architecture where each stage of the pipeline, including preprocessing, fuzzy matching, class mapping, and final consolidation, is fully documented and auditable. This structure allows investigators to tailor the pipeline to their respective data/study/field-specific environments, adjust thresholds, or substitute alternative classification systems as needed. Secondly, the use of open-source tools (e.g., DER, *thefuzz*) makes the pipeline cost-effective and accessible to research teams with varying levels of technical expertise. Third, the pipeline is designed to accommodate unstructured, self-reported medication data, common in cohort and registry studies that often lack standardized input formats. Fourth, the final output simplifies downstream analysis by collapsing medication names into binary indicators of therapeutic class, enabling integration with other covariates in epidemiological modeling. Finally, it effectively resolves brand-generic discrepancies, identifies combination drugs, and minimizes manual review through a reusable, adjudicated reference dictionary, enhancing scalability, reproducibility, and potential for multi-site harmonization.

Beyond its methodological contribution, this harmonization framework offers practical utility across multiple domains. In research settings, it can be integrated into REDCap-based or registry studies to produce reproducible, analysis-ready medication datasets for examining polypharmacy, treatment adherence, and drug–disease relationships. In clinical contexts, the pipeline can serve as a preprocessing tool to align self-reported medication lists with EHR data, supporting medication reconciliation and pharmacovigilance efforts. From a data management perspective, its modular, open-source architecture allows seamless adaptation for automated data cleaning and integration workflows across multi-site studies, thereby enhancing interoperability, transparency, and scalability.

However, we acknowledge several limitations. First, although the pipeline resolved over 94.2% of entries, as with other automated and semi-automated processes, this method’s performance depends on the quality of initial inputs. Both standardization (Step 1) and therapeutic class mapping (Step 2) utilized a fuzzy matching strategy with a similarity threshold of 90% or higher. While effective in our dataset, this threshold should be adjusted based on the quality of other datasets to which it is applied. Lowering the similarity threshold may reduce unresolved entries to under 6% while increasing the risk of misclassifications, especially for noisy datasets. Many unmatched entries resulted from spelling errors, underscoring the importance of implementing improved data entry practices, such as autocomplete tools or real-time validation at data entry.

Second, the time-consuming quality assurance process involving study-affiliated pharmacists and physicians’ oversight was used during development. Severely truncated or ambiguous entries may still require adjudication in future datasets. When spelling is accurate, the pipeline effectively distinguishes between brand and generic names through its integrated reference dictionary, thereby minimizing the need for expert review. The pipeline’s configuration was tailored to the structure and quality of our dataset, and adaptations are encouraged for use in other research settings with potentially different naming conventions or entry formats. Third, the pipeline excludes vitamins, supplements, and OTC drugs, which may limit applicability for studies examining total medication burden or self-medication behaviors. Fourth, this pipeline was designed to harmonize unstructured medication names into standardized therapeutic classes and does not currently extract dosage, frequency, route, or administration timing. This limits its ability to support analyses requiring detailed pharmacologic exposure, such as dose–response evaluation or drug–drug interaction assessment.

Fifth, this study utilized data from a single U.S.-based cohort (the DRIVES Project), which may limit generalizability to more diverse or international populations. However, the pipeline itself is data-agnostic and relies entirely on open-source tools and standardized pharmacologic vocabularies (e.g., AHFS, DrugBank, RxNorm), allowing straightforward adaptation to other datasets and regions. Sixth, the current study did not integrate EHR or pharmacy-fill data. This design decision reflects the pipeline’s primary objective: to establish a reproducible, scalable framework for harmonizing unstructured, self-reported medication data, independent of external databases. Integrating EHR data at this stage could have reduced participant willingness and increased attrition, particularly among underrepresented and minority populations, where concerns about data privacy and institutional trust are well-documented. By maintaining independence from clinical data systems, the pipeline remains broadly applicable to community-based and low-resource research settings, preserving inclusivity while ensuring reproducibility.

Seventh, the present pipeline was developed and validated exclusively for English-language data. Because it relies on English-based reference vocabularies (e.g., DrugBank, RxNorm, AHFS) and language-specific text-processing libraries, it is not currently optimized for multilingual implementation. Extending the framework to other languages would require substantial adaptation, including integration of local drug dictionaries and multilingual Named Entity Recognition (NER) models. Finally, while collapsing medications into binary therapeutic class indicators aids dimensionality reduction, it does not capture dose, frequency, route of administration, or potential drug-drug interactions. Although combination drugs were accounted for and matched during mapping, accurately assigning each component to its respective therapeutic class proved challenging. Combination drugs were classified by their primary active ingredient, which may obscure secondary pharmacologic effects.

Future research should focus on expanding this pipeline to include dosage, route, and frequency information, which can be recorded inconsistently and require more sophisticated NLP techniques for extraction and standardization. Integration with EHR data, pharmacy fill records, or claims databases would also enhance medication exposure validation and reduce reliance on self-reported data. Additionally, implementing user-facing enhancements in REDCap, such as autocomplete fields, standardized dropdowns, or real-time validation, could prevent many of the input issues we observed and reduce downstream cleaning requirements. Fine-tuned large language models (LLMs) may also be employed to improve complex spelling correction and semantic disambiguation once reproducibility and privacy safeguards are fully established. Because current proprietary and frequently updated architectures limit transparency and version stability, such models would require robust version control, frozen weights, and reproducibility protocols before integration into regulated biomedical research workflows. Adapting this pipeline to multilingual contexts, testing it across other health systems, and implementing it for more diverse, international cohorts will be crucial to demonstrating our method’s generalizability and scalability.

In conclusion, we developed a reproducible pipeline that transforms free-text medication data collected via REDCap into structured, analysis-ready variables classified by therapeutic class. By combining automated tools, pharmacist and clinician oversight, and transparent workflows, this protocol addresses a critical gap in the harmonization of research data. It offers a practical and scalable solution to one of the most persistent barriers in real-world clinical and registry research: the unstructured and unreliable nature of medication data. We believe this framework will enable more rigorous, replicable, and interpretable analyses of medication exposure across a broad range of studies on aging, dementia, and chronic diseases.

## Supplementary Information

Below is the link to the electronic supplementary material.

Supplementary Material 1

## Data Availability

The participant-level data used in this study are not publicly available due to institutional and ethical restrictions. However, the complete code for the medication data harmonization pipeline, including all steps for preprocessing, standardization, and classification, is available from the corresponding author upon reasonable request.

## Abbreviations

AHFS: American Hospital Formulary Service
AI: Artificial Intelligence
ASHP: American Society of Health-System Pharmacists
ATC: Anatomical Therapeutic Chemical classification system
CDR: Clinical Dementia Rating
DER: Drug Named Entity Recognition (Python library)
DRIVES: Driving Real-world In-Vehicle Evaluation System
EHR: Electronic Health Record
IRB: Institutional Review Board
NIA: National Institute on Aging
NIH: National Institutes of Health
NLP: Natural Language Processing
OTC: Over-the-Counter
QA: Quality Assurance
REDCap: Research Electronic Data Capture
RxNorm: Normalized Names for Clinical Drugs (U.S. National Library of Medicine)
