# Supplementary material for: Harmonizing self-reported and free text medication data: a reproducible pipeline for gerontological research
Source: BMC Med Inform Decis Mak. 2025 Dec 31;26:35. doi: 10.1186/s12911-025-03332-w (PMC12865965; doi:10.1186/s12911-025-03332-w)
Supplement: Supplementary file 1 — Supplementary Material 1 [file 12911_2025_3332_MOESM1_ESM.docx]

**Supplementary Table 1:** Common Issues Identified During Medication Data Cleaning and Harmonization

| **Issue Type** | **Description** | **Incorrect Example** | **Corrected Entry** | **Notes** |
| --- | --- | --- | --- | --- |
| Brand vs. Generic Confusion | Entries included brand names instead of generic names. | “Lipitor”, “Glucophage” | “Atorvastatin”, “Metformin” | Brand-generic mapping described in drug curation workflow. |
| Capitalization Variants | Inconsistent casing across entries. | “ATORVASTATIN”, “lisinopril” | “Atorvastatin”, “Lisinopril” | Mentioned in harmonization steps for standardization. |
| Combination Drugs | Multi-ingredient medications required disaggregation. | “Janumet”, “Amlodipine/Benazepril” | “Sitagliptin + Metformin”, “Amlodipine + Benazepril” | Directly addressed in harmonization and curation sections. |
| Dosage Embedded in Name | Strengths or doses embedded with drug name. | “Metformin 500mg”, “Aspirin 81 mg” | “Metformin”, “Aspirin” | Noted as a common formatting issue; dose separated during extraction. |
| No Match Found / Ambiguous Entries | Entries lacking identifiable names or drug info. | “Blood pressure med”, “Insulin” | Flagged as ambiguous/unclassifiable | Protocol specifies that such entries were marked and excluded. |
| Route Included in Name | Entries contained administration route. | “Timolol eye drops” | “Timolol” | Route removed for standardization; confirmed in drug flag section. |
| Spelling Errors | Misspelled drug names noted during manual review. | “Metmorphin”, “Losarton” | “Metformin”, “Losartan” | Explicitly mentioned in protocol under common entry problems. |
| Supplements | Vitamin/mineral/supplemental therapies variably entered and flagged. | “Vitamin D”, “Omega 3”, “B-12” | “Vitamin D”, “Omega-3”, “Vitamin B12” | Defined as a separate class; excluded from primary exposure models. |

This table outlines recurring issues encountered during the medication data curation process, including spelling errors, brand vs. generic confusion, capitalization inconsistency, and ambiguous entries. Each issue is accompanied by an example, corrected entry, and procedural notes based on the harmonization protocol and Research Electronic Data Capture (REDCap) database review.

- All issues listed above were explicitly described or inferred from procedural steps in the harmonization protocol.
- Correction and validation followed RxNorm mapping, manual review, and class curation based on ATC principles.
- These categories reflect actual entries in the REDCap database used in the study.

**Supplementary Table 2:** Therapeutic Classes with Their Mapped Medications

This table provides a comprehensive list of 65 therapeutic classes and the corresponding medications mapped under each category. It includes drugs from diverse pharmacological categories such as cardiovascular agents, CNS^b^ drugs, antidiabetics, antidepressants, antihistamines, antibiotics, and more. Abbreviations for major therapeutic categories are defined for clarity.

| **S. No.^a^** | **Therapeutic Class** | **Medication Name(s)** |
| --- | --- | --- |
| 1 | Alpha Blockers | Carvedilol, Doxazosin, Fludrocortisone, Prazosin, Terazosin |
| 2 | Antacids & Adsorbents | Bismuth subsalicylate, Gaviscon, Simethicone |
| 3 | Anti-Infectives | Acyclovir, Amoxicillin, Azelaic acid, Azithromycin, Cefdinir, Cefotetan, Cephalexin, Chlorhexidine, Ciclopirox, Ciprofloxacin, Clindamycin, Clotrimazole, Dapsone, Dolutegravir, Doravirine, Doxycycline, Econazole, Famciclovir, Hydroxychloroquine, Ketoconazole, Levofloxacin, Mefloquine, Methenamine, Metronidazole, Moxifloxacin, Mupirocin, Nitrofurantoin, Nystatin, Ofloxacin, Rifampin, Spironolactone, Sulfamethoxazole, Terbinafine, Tetracycline, Valacyclovir, Valganciclovir |
| 4 | Anticonvulsants | Carbamazepine, Clonazepam, Gabapentin, Lamotrigine, Levetiracetam, Pregabalin, Primidone, Topiramate, Zonisamide |
| 5 | Antidepressants | Amitriptyline, Amoxapine, Bupropion, Citalopram, Desvenlafaxine, Doxepin, Duloxetine, Escitalopram, Fluoxetine, Imipramine, Mirtazapine, Nortriptyline, Paroxetine, Phenelzine, Sertraline, Trazodone, Venlafaxine, Vilazodone |
| 6 | Antidiabetics | Alogliptin, Canagliflozin, Dapagliflozin, Dulaglutide, Empagliflozin, Exenatide, Glimepiride, Glipizide, Insulin glargine, Insulin lispro, Linagliptin, Liraglutide, Metformin, Pioglitazone, Saxagliptin, Semaglutide, Sitagliptin, Tirzepatide |
| 7 | Antidiarrheal Agents | Bismuth subsalicylate, Loperamide |
| 8 | Antiemetics | Granisetron, Meclizine, Ondansetron, Prochlorperazine |
| 9 | Antihistamines | Azelastine, Cetirizine, Cimetidine, Cyproheptadine, Diphenhydramine, Doxylamine, Famotidine, Fexofenadine, Hydroxyzine, Ketotifen, Levocetirizine, Loratadine, Olopatadine |
| 10 | Antimigraine Agents | Almotriptan, Eletriptan, Ergotamine, Naratriptan, Rizatriptan, Sumatriptan, Zolmitriptan |
| 11 | Antimuscarinics / Antispasmodics | Dicyclomine, Oxybutynin, Tolterodine, Trospium |
| 12 | Antineoplastic Agents | Anastrozole, Letrozole, Tamoxifen |
| 13 | Antiparkinson | Amantadine, Benztropine, Bromocriptine, Carbidopa, Entacapone, Levodopa, Pramipexole, Rasagiline, Ropinirole, Selegiline |
| 14 | Antipsychotics | Aripiprazole, Asenapine, Cariprazine, Chlorpromazine, Clozapine, Haloperidol, Iloperidone, Loxapine, Lurasidone, Mesoridazine, Olanzapine, Paliperidone, Perphenazine, Pimozide, Prochlorperazine, Quetiapine, Risperidone, Thioridazine, Thiothixene, Trifluoperazine, Ziprasidone |
| 15 | Antitussives | Benzonatate, Dextromethorphan, Hydrocodone |
| 16 | Autonomic Drugs | Clonidine, Guanfacine, Labetalol, Methyldopa, Metoprolol, Propranolol |
| 17 | Benzodiazepines | Alprazolam, Chlordiazepoxide, Clonazepam, Diazepam, Estazolam, Flurazepam, Lorazepam, Midazolam, Temazepam, Triazolam |
| 18 | Beta Blockers | Atenolol, Bisoprolol, Carvedilol, Labetalol, Metoprolol, Nadolol, Nebivolol, Propranolol, Sotalol |
| 19 | Bisphosphonates | Alendronate, Ibandronate, Risedronate |
| 20 | Central Nervous System (CNS) Drugs | Lmotriptan, Alprazolam, Amitriptyline, Amoxapine, Amphetamine, Aripiprazole, Armodafinil, Aspirin, Baclofen, Benztropine, Bupropion, Buspirone, Butalbital, Carbamazepine, Carbidopa, Carisoprodol, Celecoxib, Chlorpromazine, Citalopram, Clonazepam, Codeine, Cyclobenzaprine, Desvenlafaxine, Dextroamphetamine, Diazepam, Diclofenac, Diphenhydramine, Divalproex, Donepezil, Doxepin, Duloxetine, Escitalopram, Fentanyl, Fluoxetine, Gabapentin, Haloperidol, Hydrocodone, Hydromorphone, Hydroxyzine, Ibuprofen, Imipramine, Indomethacin, Lamotrigine, Levetiracetam, Lidocaine, Lithium, Lorazepam, Lurasidone, Meclizine, Meloxicam, Memantine, Methadone, Methocarbamol, Methylphenidate, Mirtazapine, Modafinil, Morphine, Naproxen, Nortriptyline, Olanzapine, Oxcarbazepine, Oxycodone, Paroxetine, Phenytoin, Pregabalin, Prochlorperazine, Promethazine, Quetiapine, Rasagiline, Risperidone, Rivastigmine, Rizatriptan, Ropinirole, Sertraline, Sumatriptan, Temazepam, Thioridazine, Thiothixene, Tizanidine, Topiramate, Tramadol, Trazodone, Triazolam, Valproic acid, Venlafaxine, Vilazodone, Zaleplon, Ziprasidone, Zolpidem |
| 21 | CNS Stimulants | Amphetamine, Dextroamphetamine, Lisdexamfetamine, Methylphenidate, Modafinil |
| 22 | Cardiovascular (CVD) Drugs | Alfuzosin, Alirocumab, Aliskiren, Amiodarone, Amlodipine, Atenolol, Atorvastatin, Benazepril, Bisoprolol, Carvedilol, Chlorthalidone, Clonidine, Clopidogrel, Dabigatran, Digoxin, Diltiazem, Doxazosin, Enalapril, Enoxaparin, Ezetimibe, Felodipine, Fenofibrate, Furosemide, Gemfibrozil, Hydrochlorothiazide, Indapamide, Irbesartan, Isosorbide dinitrate, Isosorbide mononitrate, Labetalol, Lisinopril, Losartan, Lovastatin, Metoprolol, Nebivolol, Niacin, Nifedipine, Nitroglycerin, Pentoxifylline, Pravastatin, Propranolol, Quinapril, Ramipril, Rivaroxaban, Rosuvastatin, Simvastatin, Spironolactone, Terazosin, Timolol, Torsemide, Triamterene, Valsartan, Warfarin |
| 23 | Calcium Channel Blockers | Amlodipine, Diltiazem, Felodipine, Nifedipine, Verapamil |
| 24 | Cardiotonic / Antiarrhythmics | Amiodarone, Digoxin, Dofetilide, Flecainide, Propafenone, Quinidine, Sotalol |
| 25 | Central Alpha Agonists / Direct Vasodilators | Clonidine, Hydralazine, Minoxidil |
| 26 | Cholinergic Agents | Bethanechol, Neostigmine, Physostigmine, Pyridostigmine |
| 27 | Disease-Modifying Antirheumatic Drugs (DMARDs)^h^ | Abatacept, Adalimumab, Anakinra, Azathioprine, Certolizumab, Cyclosporine, Etanercept, Golimumab, Hydroxychloroquine, Infliximab, Leflunomide, Methotrexate, Mycophenolate mofetil, Rituximab, Sulfasalazine, Tacrolimus, Tocilizumab, Tofacitinib, Ustekinumab |
| 28 | EENT^i^ (Eye, Ear, Nose, Throat) | Azelastine, Brimonidine, Dorzolamide, Latanoprost, Olopatadine, Timolol, Travoprost |
| 29 | Electrolytes / Diuretics / Water Balance | Calcium acetate, Chlorthalidone, Furosemide, Hydrochlorothiazide, Indapamide, Metolazone, Potassium chloride, Spironolactone |
| 30 | Expectorants | Guaifenesin |
| 31 | First-Generation Antidepressants | Amitriptyline, Amoxapine, Clomipramine, Doxepin, Imipramine, Maprotiline, Nortriptyline, Protriptyline, Trimipramine |
| 32 | First-Generation Antihistamines | Brompheniramine, Chlorpheniramine, Cyproheptadine, Diphenhydramine, Doxylamine, Hydroxyzine, Meclizine, Promethazine, Triprolidine |
| 33 | Gastrointestinal (GI)^j^ Drugs | Bismuth subsalicylate, Dicyclomine, Esomeprazole, Famotidine, Lansoprazole, Metoclopramide, Omeprazole, Pantoprazole, Ranitidine, Sucralfate |
| 34 | Gout Medications | Allopurinol, Colchicine, Probenecid |
| 35 | H2 Antagonists | Cimetidine, Famotidine, Nizatidine, Ranitidine |
| 36 | Herbal & Dietary Supplements | Black cohosh, Coenzyme q10, Cranberry, Echinacea, Fish oil, Garlic, Ginkgo, Ginseng, Glucosamine, Melatonin, Omega-3 acid, Saw palmetto, Soy isoflavones, St, John's wort, Vitamin c |
| 37 | Incretin Mimetics | Dulaglutide, Exenatide, Liraglutide, Semaglutide |
| 38 | Insulin | Insulin aspart, Insulin detemir, Insulin glargine, Insulin lispro, Insulin nph, Insulin regular |
| 39 | Laxatives / Cathartics | Bisacodyl, Docusate, Lactulose, Magnesium citrate, Magnesium hydroxide, Polyethylene glycol, Psyllium, Senna, Sodium phosphate |
| 40 | Leukotriene Modifiers | Montelukast, Zafirlukast, Zileuton |
| 41 | Lipid-Lowering Agents | Atorvastatin, Cholestyramine, Colesevelam, Colestipol, Ezetimibe, Fenofibrate, Gemfibrozil, Lovastatin, Niacin, Pravastatin, Rosuvastatin, Simvastatin |
| 42 | MAO^l^ Inhibitors | Isocarboxazid, Phenelzine, Selegiline, Tranylcypromine |
| 43 | Miscellaneous Drugs | Conjugated estrogens, Dantrolene, Finasteride, Medroxyprogesterone, Mesalamine, Methenamine, Mirabegron, Phenazopyridine, Varenicline |
| 44 | Non-Benzodiazepine Hypnotics/Sedatives | Eszopiclone, Ramelteon, Suvorexant, Zaleplon, Zolpidem |
| 45 | NSAIDs^e^ | Acetaminophen, Aspirin, Celecoxib, Diclofenac, Ibuprofen, Indomethacin, Meloxicam, Naproxen, Piroxicam |
| 46 | Opioids | Buprenorphine, Codeine, Fentanyl, Hydrocodone, Hydromorphone, Methadone, Morphine, Nalbuphine, Oxycodone, Tapentadol, Tramadol |
| 47 | Proton Pump Inhibitors (PPIs)^f^ | Dexlansoprazole, Esomeprazole, Lansoprazole, Omeprazole, Pantoprazole, Rabeprazole |
| 48 | Prokinetics | Metoclopramide |
| 49 | RAAS^g^ Inhibitors | Benazepril, Captopril, Enalapril, Fosinopril, Lisinopril, Losartan, Olmesartan, Perindopril, Quinapril, Ramipril, Telmisartan, Valsartan |
| 50 | Respiratory Drugs | Albuterol, Budesonide, Fluticasone, Ipratropium, Levalbuterol, Mometasone, Montelukast, Salmeterol, Tiotropium |
| 51 | Second-Generation Antidepressants | Bupropion, Desvenlafaxine, Duloxetine, Escitalopram, Fluoxetine, Mirtazapine, Paroxetine, Sertraline, Trazodone, Venlafaxine, Vilazodone, Vortioxetine |
| 52 | Second-Generation Antihistamines | Cetirizine, Desloratadine, Fexofenadine, Levocetirizine, Loratadine |
| 53 | Skeletal Muscle Relaxants | Baclofen, Carisoprodol, Chlorzoxazone, Cyclobenzaprine, Dantrolene, Methocarbamol, Metaxalone, Orphenadrine, Tizanidine |
| 54 | SSRIs^c^ / SNRIs^d^ | Citalopram, Desvenlafaxine, Duloxetine, Escitalopram, Fluoxetine, Paroxetine, Sertraline, Venlafaxine, Vilazodone, Vortioxetine |
| 55 | Steroids | Budesonide, Clobetasol, Dexamethasone, Fluticasone, Hydrocortisone, Methylprednisolone, Mometasone, Prednisolone, Prednisone, Triamcinolone |
| 56 | Sympathomimetic Agents | Albuterol, Levalbuterol, Salmeterol |
| 57 | Tricyclic Antidepressants (TCAs)^k^ | Amitriptyline, Amoxapine, Clomipramine, Desipramine, Doxepin, Imipramine, Nortriptyline, Protriptyline, Trimipramine |
| 58 | Thyroid Agents | Levothyroxine, Liothyronine, Methimazole, Propylthiouracil |
| 59 | Topical Skin Preparations | Bacitracin, Clobetasol, Dexamethasone, Hydrocortisone, Mometasone, Mupirocin, Neomycin, Nystatin, Triamcinolone |
| 60 | Vasodilating Agents | Hydralazine, Isosorbide dinitrate, Isosorbide mononitrate, Minoxidil, Nitroglycerin, Sildenafil |
| 61 | Vitamins | Calcitriol, Cholecalciferol, Cyanocobalamin, Ergocalciferol, Folic acid |
| 62 | Anticoagulants / Antiplatelets / Anti-thrombolytics / Antianemia Agents | Apixaban, Clopidogrel, Dabigatran, Enoxaparin, Epoetin alfa, Ferrous sulfate, Fondaparinux, Rivaroxaban, Warfarin |
| 63 | GI Antispasmodics | Dicyclomine, Hyoscyamine |
| 64 | Antiplatelets | Aspirin, Clopidogrel, Dipyridamole, Ticagrelor |
| 65 | Miscellaneous | Sodium polystyrene sulfonate, Urea |

**Abbreviations**

1. ***S. No.*** – Serial Number
2. ***CNS*** – Central Nervous System
3. ***SSRI*** – Selective Serotonin Reuptake Inhibitor
4. ***SNRI*** – Serotonin-Norepinephrine Reuptake Inhibitor
5. ***NSAIDs*** – Nonsteroidal Anti-Inflammatory Drugs
6. ***PPIs*** – Proton Pump Inhibitors
7. ***RAAS*** – Renin-Angiotensin-Aldosterone System
8. ***DMARDs*** – Disease-Modifying Antirheumatic Drugs
9. ***EENT*** – Eye, Ear, Nose, and Throat
10. ***GI*** – Gastrointestinal
11. ***TCAs*** – Tricyclic Antidepressants
12. ***MAO*** – Monoamine Oxidase

**Supplementary Table 3: Audit Log of Medication Harmonization: Classification Reassignment and Merging Decisions for Analytical Consistency**

**Section 1: Class Merges and Modifications**

This table documents the reclassification of selected therapeutic classes to improve consistency and clinical relevance. It includes the original and updated classifications, rationale for each change, standardized generic names, AHFS therapeutic class, mapped disease indications, reviewer details, and final decisions. A study-affiliated pharmacist and physicians made reclassifications to resolve redundancy, align with pharmacological mechanisms, and ensure accurate disease mapping.

| **Original Classification** | **New Classification** | **Rationale** | **Standardized Generic Name** | **AHFS Class** | **Disease Mapping** | **Reviewed By** | **Final Decision** | **Notes** |
| --- | --- | --- | --- | --- | --- | --- | --- | --- |
| Antiplatelets | Anticoagulants / Antiplatelets / Antithrombolytics / Antianemia | Merged to represent comprehensive anticoagulant therapy, ensuring all agents affecting hemostasis are captured. | Clopidogrel, Warfarin, Rivaroxaban | Antithrombotic Agents | Cardiovascular disease, stroke prevention | Study-affiliated Pharmacist + Physicians | Merge approved | Full class name retained for clarity |
| Central alpha agonists / Direct vasodilators | Vasodilators | Both groups act via vasodilation to lower blood pressure and improve cardiac output. | Clonidine, Hydralazine | Vasodilating Agents | Hypertension, heart failure | Study-affiliated Pharmacist + Physicians | Merge approved | Used consistently in CVD grouping |
| Corticosteroids + Adrenals/Steroids | Steroids | These medications share anti-inflammatory properties and are used primarily for inflammatory and autoimmune conditions. No anabolic or sex steroids included. | Prednisone, Dexamethasone, Hydrocortisone | Steroids | Inflammatory diseases (e.g., asthma, arthritis) | Study-affiliated Pharmacist + Physicians | Merge approved | Grouped to eliminate subclass redundancy |
| Hormones and Synthetic Substitutes | Reassigned to relevant classes | Non-cohesive group; drugs reassigned to primary therapeutic classes (e.g., thyroid agents, Steroids). | Liothyronine, Cabergoline | Thyroid Agents, Miscellaneous | Hypothyroidism, hyperprolactinemia | Study-affiliated Pharmacist + Physicians | Class dissolved | Manually redistributed |
| Oral Antidiabetics | Antidiabetics | Route of administration not relevant for class inference; drugs reassigned by mechanism. | Metformin, Glipizide | Antidiabetic Agents | Type 2 Diabetes Mellitus | Study-affiliated Pharmacist + Physicians | Class dissolved | Collapsed to simplify classification |

- **Original Classification:** Medication category as extracted from the REDCap dataset.
- **New Classification:** Consolidated class name used for final analysis.
- **Rationale:** Clinical justification for class merging, including mechanistic or therapeutic overlap.
- **Standardized Generic Name:** Examples of generic drugs representing each merged class.
- **AHFS Class:** Corresponding category based on the American Hospital Formulary Service classification.
- **Disease Mapping:** Primary clinical indications for which the merged drug class is prescribed.
- **Reviewed By:** Indicates internal clinical validation of each classification decision.
- **Final Decision:** Outcome of the classification review process (e.g., "Merge approved", "Class dissolved").
- **Notes:** Additional details regarding subclass adjustments, exclusions, or exceptions.
- *CVD* – Cardiovascular Disease

**Section 2: Individual Medication Class Reassignments and Merging Rationale**

This table presents the reclassification of medications originally labeled in the REDCap database, with updates to standardized therapeutic classes based on pharmacologic mechanism, clinical use, and analytical consistency. Each row includes the original and new classification, rationale for reclassification, representative standardized generic names, AHFS therapeutic class, mapped disease indications, reviewer details, final decision, and relevant notes. All medication reclassifications were thoroughly reviewed and approved by the study-affiliated pharmacist and physician team: Kebede Beyene, PhD (Pharmacist); David B. Carr, MD; Ramkrishna K. Singh, MBBS, MPH; and Semere Bekena, MD, MPH (Physicians) to ensure accuracy for cognition-related phenotypic analyses.

| **Original Classification** | **New Classification** | **Rationale** | **Standardized Generic Name** | **AHFS Class** | **Disease Mapping** | **Review Notes** |
| --- | --- | --- | --- | --- | --- | --- |
| Ado-trastuzumab emtansine | Antineoplastic Agents | Antibody-drug conjugate for HER2+ breast cancer; reassigned to oncology drugs. | Ado-trastuzumab emtansine | Antineoplastics | Breast cancer | Reassigned.  Cancer-specific targeting. |
| Allopurinol | Gout medications | Uric acid-lowering therapy for gout; better represented under a dedicated class. | Allopurinol | Antigout Agents | Gout, hyperuricemia | Reassigned.  Harmonized with other antigout drugs. |
| Amantadine | CNS Agents | NMDA antagonist used in Parkinsonism and dyskinesia; reassigned from antivirals. | Amantadine | Antiparkinsonian Agents | Parkinson’s disease, drug-induced extrapyramidal symptoms | Reassigned.  Primary indication is neurological. |
| Apomorphine | CNS Agents | Dopamine agonist for Parkinson's "off" episodes; reassigned from general neurology. | Apomorphine | Antiparkinsonian Agents | Parkinson’s disease | Reassigned.  More specific than general dopaminergic class. |
| Cabergoline | Miscellaneous | Dopamine agonist; lacks sufficient representation in other classes. | Cabergoline | Dopamine Agonists | Hyperprolactinemia, Parkinson’s | Reassigned.  No merged subclass suitable. |
| Cholecalciferol | Vitamins | A nutritional supplement not used as a pharmacologic agent; classified under vitamins. | Cholecalciferol | Vitamins | Vitamin D deficiency | Reassigned.  Removed from Miscellaneous. |
| Cinacalcet | Endocrine / Mineral-modifying agents | Calcimimetic for secondary hyperparathyroidism; reassigned from Miscellaneous. | Cinacalcet | Endocrine Agents | Hyperparathyroidism | Reassigned.  No prior defined category. |
| Colchicine | Gout medications | Anti-inflammatory agent used in acute gout flares; misclassified under Miscellaneous. | Colchicine | Antigout Agents | Gout | Reassigned.  Clarifies therapeutic role. |
| Dehydroepi-androsterone | Hormones and Synthetic Substitutes | Reassigned from supplement category due to hormonal action. | DHEA | Hormonal Agents | Adrenal insufficiency | Reassigned.  Addressed therapeutic mechanism. |
| Denosumab | Bone-Modifying Agents | RANKL inhibitor for osteoporosis and bone metastases; pulled from Oncology. | Denosumab | Osteoporosis Agents | Osteoporosis, bone loss | Reassigned.  Not exclusive to cancer care. |
| Dexamethasone | Steroids | Strong glucocorticoid; aligned with corticosteroid class. | Dexamethasone | Steroids | Inflammation, immunosuppression | Reassigned.  Anti-inflammatory mechanism. |
| Droxidopa | Cardiovascular Agents | Norepinephrine precursor for neurogenic orthostatic hypotension; reassigned from Miscellaneous. | Droxidopa | Vasopressors | Neurogenic orthostatic hypotension | Reassigned.  Mechanistically distinct but clear cardiovascular target. |
| Estradiol | Hormonal Agents | Sex hormone replacement agent; reassigned from Miscellaneous to hormone class. | Estradiol | Estrogens | Menopausal symptoms, osteoporosis prevention | Reassigned.  Consistent with endocrine therapy. |
| Evolocumab | Lipid-lowering agents | Correct class assignment due to its LDL-lowering role. | Evolocumab | Antihyperlipidemic Agents | Dyslipidemia | Reassigned.  Consistent with other PCSK9 inhibitors. |
| Febuxostat | Gout medications | Uric acid synthesis inhibitor; better grouped with gout-related agents. | Febuxostat | Antigout Agents | Gout | Reassigned.  Gout-specific mechanism. |
| Finasteride | Hormonal Agents | 5-alpha reductase inhibitor; used in androgen-sensitive conditions. | Finasteride | Androgen Inhibitors | BPH, alopecia | Reassigned.  Clarifies anti-androgen mechanism. |
| Finasteride | Urologic Agents | 5-alpha-reductase inhibitor for BPH; moved from Hormonal class to Urology-focused category. | Finasteride | BPH Agents | Benign prostatic hyperplasia | Reassigned.  Matches specialty prescribing patterns. |
| Glatiramer | MS-treatment drugs | Specific immunomodulatory agent used in multiple sclerosis; not appropriately captured under Miscellaneous. | Glatiramer acetate | Immunomodulatory Agents | Multiple sclerosis | Reassigned.  Ensures therapeutic accuracy. |
| Ibandronate | Bisphosphonates | Correctly reclassified for alignment with bisphosphonate class. | Ibandronate | Bone Resorption Inhibitors | Osteoporosis | Reassigned.  Supports bone health categorization. |
| Inclisiran | Lipid-lowering agents | PCSK9 inhibitor with direct lipid-lowering effects; misclassified initially. | Inclisiran | Antihyperlipidemic Agents | Hypercholesterolemia | Reassigned.  Enhances CVD class representation. |
| Lactulose | Gastrointestinal Agents | Osmotic laxative for hepatic encephalopathy; reassigned for GI/hepatic clarity. | Lactulose | Laxatives | Hepatic encephalopathy, constipation | Reassigned.  Better reflects pharmacologic action. |
| Leuprolide | Hormonal / Oncologic Agents | GnRH agonist; reassigned from Oncology-only to dual category recognizing hormonal therapy in both cancer and endocrine conditions. | Leuprolide | Antineoplastics / Hormonal Agents | Prostate cancer, endometriosis | Reassigned.  Reflects dual-use across specialties. |
| Linaclotide | Laxatives / Cathartics | Guanylate cyclase-C agonist used in chronic constipation; reassigned from Miscellaneous. | Linaclotide | Laxatives | IBS-C, chronic idiopathic constipation | Reassigned  Mechanism-specific realignment. |
| Linaclotide | Gastrointestinal Agents | Guanylate cyclase-C agonist for IBS-C; previously under miscellaneous. | Linaclotide | Laxatives / Secretagogues | IBS-C, chronic constipation | Reassigned.  GI-specific with unique MoA. |
| Linoleic Acid | Herbs and Supplements | Essential fatty acid not classified as lipid-lowering drug. | Linoleic Acid | Nutritional Supplements | Nutritional deficiency | Reassigned.  Corrected functional role. |
| Liothyronine | Thyroid Agents | Reassigned from hormone group due to direct thyroid hormone replacement use. | Liothyronine | Thyroid Hormones | Hypothyroidism | Reassigned.  Better analytic utility |
| Liraglutide | Antidiabetic / Cardiometabolic Agents | GLP-1 analog; reassigned from general endocrine to dual-use in diabetes and weight management. | Liraglutide | Incretin Mimetics | T2DM, obesity | Reassigned.  Reflects evolving therapeutic guidelines. |
| Metolazone | Diuretics | Loop-sparing thiazide-like diuretic; grouped under Diuretics from Antihypertensives. | Metolazone | Diuretics | CHF, hypertension | Reassigned.  Reflects mechanism and synergy. |
| Midodrine | Cardiovascular Agents | Alpha agonist for orthostatic hypotension; reclassified for clearer cardiovascular use. | Midodrine | Vasopressors | Orthostatic hypotension | Reassigned.  Clarifies vascular mechanism. |
| Obeticholic acid | Gastrointestinal Drugs | FXR agonist used for liver-related GI conditions; misclassified. | Obeticholic acid | Hepatoprotective Agents | Primary biliary cholangitis | Reassigned.  GI system relevance highlighted. |
| Patiromer | Electrolyte-Modifying Agents | Used to treat hyperkalemia; reassigned from Miscellaneous. | Patiromer | Potassium Binders | Hyperkalemia | Reassigned.  Electrolyte correction, renal relevance. |
| Plecanatide | Laxatives / Cathartics | Mechanism similar to Linaclotide; realigned to appropriate GI class. | Plecanatide | Laxatives | IBS-C | Reassigned.  Promotes class-level inference. |
| Ranolazine | Antianginal Agents | Antianginal agent with metabolic effects; moved from vasodilators. | Ranolazine | Antianginal Agents | Chronic angina | Reassigned.  Better classified under anti-ischemic therapy. |
| Rifaximin | Gastrointestinal Agents | Oral non-absorbable antibiotic used primarily for hepatic encephalopathy; removed from systemic antibiotic class. | Rifaximin | Antibiotics / GI Agents | Hepatic encephalopathy, IBS-D | Reassigned.  Emphasizes local GI mechanism. |
| Rizatriptan | Antimigraine Agents | Triptan-class drug; reassigned from vasodilators to target-specific group. | Rizatriptan | Antimigraine Agents | Migraine | Reassigned.  Improves pharmacologic clarity |
| Sevelamer | Electrolyte / Renal Agents | Phosphate binder for chronic kidney disease; moved from Miscellaneous to Renal category. | Sevelamer | Phosphate Binders | CKD-mineral bone disorder | Reassigned.  Used in ESRD-specific indications. |
| Sodium Bicarbonate | Electrolyte / Acid-Base Agents | Buffers systemic acidosis; reclassified to emphasize metabolic role. | Sodium Bicarbonate | Alkalinizing Agents | Metabolic acidosis | Reassigned.  Differentiated from GI antacids. |
| Tacrolimus | Topical / Skin Preparation | Topical immune suppressant used in dermatology; better placed in skin preparation class. | Tacrolimus | Dermatologic Agents | Eczema, dermatitis | Reassigned.  Clarifies mode and route. |
| Tadalafil | Urologic Agents | Used in erectile dysfunction and BPH; reassigned from vasodilators. | Tadalafil | Genitourinary Agents | Erectile dysfunction, BPH | Reassigned.  Therapeutic use emphasized over mechanism. |
| Teriparatide | Bone-modifying agents | Parathyroid analog for osteoporosis; removed from Miscellaneous. | Teriparatide | Parathyroid Hormones | Osteoporosis | Reassigned.  Improved alignment with therapeutic class. |
| Tolvaptan | Renal Agents | Vasopressin antagonist used in hyponatremia and ADPKD; reassigned from Endocrine. | Tolvaptan | ADH Antagonists | Hyponatremia, polycystic kidney disease | Reassigned.  Precision in kidney-related classification. |
| Ursodiol | Hepatobiliary Agents | Used for gallstone dissolution and primary biliary cholangitis; reassigned from Miscellaneous. | Ursodiol | Gallstone Dissolution Agents | Gallstones, PBC | Reassigned.  Narrow hepatobiliary indication. |
| Valganciclovir | Antiviral Agents | Narrow antiviral for CMV in transplant settings; separated from general antimicrobials. | Valganciclovir | Antivirals | Cytomegalovirus infection | Reassigned.  Specific to immunocompromised populations. |
| Zoledronic acid | Bisphosphonates | Reclassified for consistency with other osteoporosis treatments. | Zoledronic acid | Bone Resorption Inhibitors | Osteoporosis, bone metastasis | Reassigned.  Previously under Miscellaneous. |

- **Original Classification:** Medication class labels extracted from the REDCap database as entered by data abstractors or participants.
- **New Classification:**  Standardized class groupings developed to improve analytical coherence and reduce subclass redundancy.
- **Rationale:**  Pharmacological, clinical, or methodological justification for class reassignments or merges.
- **Standardized Generic Name:**  Example agents representing each classification change; not exhaustive.
- **AHFS Class:**  American Hospital Formulary Service therapeutic category.
- **Disease Mapping:**  Primary indication or disease condition linked to each new class, used for phenotype alignment in cognition-focused analyses.
- **Review Notes:** Consensus outcomes of the class reassignment review, along with contextual details relevant for analytic processing and variable recoding.
- ***HER2+*** – Human Epidermal Growth Factor Receptor 2-Positive; ***CNS*** – Central Nervous System; ***NMDA*** – N-Methyl-D-Aspartate; ***DHEA*** – Dehydroepiandrosterone; ***RANKL*** – Receptor Activator of Nuclear Factor Kappa-B Ligand; ***BPH*** – Benign Prostatic Hyperplasia; ***MS*** – Multiple Sclerosis; ***PCSK9*** – Proprotein Convertase Subtilisin/Kexin Type 9; ***LDL*** – Low-Density Lipoprotein; ***CVD*** – Cardiovascular Disease; ***GI*** – Gastrointestinal; ***GnRH*** – Gonadotropin-Releasing Hormone; ***IBS-C*** – Irritable Bowel Syndrome with Constipation; ***MoA*** – Mechanism of Action; ***GLP-1*** – Glucagon-Like Peptide-1; ***T2DM*** – Type 2 Diabetes Mellitus; ***CHF*** – Congestive Heart Failure; ***FXR*** – Farnesoid X Receptor; ***IBS-D*** – Irritable Bowel Syndrome with Diarrhea; ***CKD*** – Chronic Kidney Disease; ***ESRD*** – End-Stage Renal Disease; ***ADPKD*** – Autosomal Dominant Polycystic Kidney Disease; ***CMV*** – Cytomegalovirus; ***PBC*** – Primary Biliary Cholangitis.

**Section 3: Therapeutic Class Consolidation for Aging and Dementia-Focused Pharmacological Analyses**

This table summarizes the systematic merging of pharmacologic subclasses into broader therapeutic categories to support unified medication exposure analyses in Alzheimer’s and dementia research. Each merged class lists the included subclasses and provides the clinical rationale for grouping based on shared mechanisms of action, therapeutic indications, and relevance to cognitive outcomes. All merges were reviewed and approved by a study-affiliated pharmacist and physicians to ensure classification accuracy and analytical coherence.

| **Final Merged Class** | **Included Subclasses** | **Rationale for Merging** |
| --- | --- | --- |
| **Antidepressant agents** | • First-generation antidepressants  • Second-generation antidepressants  • Selective serotonin reuptake inhibitors (SSRIs)  • Serotonin-norepinephrine reuptake inhibitors (SNRIs)  • Monoamine oxidase inhibitors (MAOIs)  • Tricyclic antidepressants (TCAs) | All included subclasses are used to treat depression and mood disorders. Grouping allows for analysis of overall antidepressant exposure, regardless of specific mechanism or generation. |
| **Antihistaminic agents** | • First-generation antihistamines  • Second-generation antihistamines | Subclasses grouped due to shared H1 receptor antagonism. Merging simplifies analysis of antihistaminic exposure and cognitive risk. |
| **Cardiovascular agents** | • Beta-blockers  • Alpha-blockers  • Calcium channel blockers  • RAAS inhibitors  • Cardiotonic/antiarrhythmic agents  • Central alpha agonists, vasodilators  • Sympatholytic agents  • Lipid-lowering agents  • Anticoagulants and antiplatelets  • Electrolytes, diuretics, fluid balance agents  • Sympathomimetic agents | All drugs included modulate cardiovascular physiology, such as blood pressure, heart rhythm, and lipid levels. Merging supports cognitive outcome analyses related to cardiovascular pharmacology. |
| **Central nervous system agents** | • Benzodiazepines  • Non-benzodiazepine hypnotics/sedatives  • CNS stimulants  • Antipsychotics  • Anticonvulsants  • Antiparkinsonian agents  • Antimigraine agents  • Skeletal muscle relaxants  • First- and second-generation antidepressants  • MAOIs, SSRIs, SNRIs, TCAs | These medications act on the central nervous system and are prescribed for psychiatric, neurologic, and cognitive conditions. Merging enables unified analysis of CNS pharmacotherapy. |
| **Gastrointestinal agents** | • Antacids and adsorbents  • H2 receptor antagonists  • Proton pump inhibitors (PPIs)  • Laxatives and cathartics  • Antidiarrheal agents  • Antiemetics  • Prokinetic agents | These medications are used for gastrointestinal disorders including reflux, ulcers, nausea, and bowel irregularities. Grouping enables analysis of GI drug exposure and cognitive links. |
| **Respiratory system agents** | • Antitussives  • Expectorants  • Leukotriene modifiers  • Smooth muscle relaxants  • Sympathomimetic agents  • First- and second-generation antihistamines  • Antimuscarinics/antispasmodics | All included drugs are used for managing respiratory conditions (e.g., asthma, COPD, allergic rhinitis). Grouping reflects shared therapeutic targets and indications. |

- This classification reflects the systematic merging of pharmacological subclasses into broader therapeutic categories to support harmonized medication exposure analyses in Alzheimer’s and dementia research.
- Merges were based on shared mechanisms, clinical indications, and relevance to cognitive outcomes, and were jointly reviewed by a study-affiliated pharmacist and physician for clinical accuracy.

**Supplementary Appendix 6: Code Availability**

The medication matching algorithm used in this study was implemented using a custom Python pipeline. The full code is openly available at the following GitHub repository:

<https://github.com/cchen-bit/Medication>
